# The *MHC2TA *-168A>G gene polymorphism is not associated with rheumatoid arthritis in Austrian patients

**DOI:** 10.1186/ar1974

**Published:** 2006-06-15

**Authors:** Babak Yazdani-Biuki, Kerstin Brickmann, Klaus Wohlfahrt, Thomas Mueller, Winfried März, Wilfried Renner, Manuela Gutjahr, Uwe Langsenlehner, Peter Krippl, Thomas C Wascher, Bernhard Paulweber, Winfried Graninger, Hans-Peter Brezinschek

**Affiliations:** 1 Department of Internal Medicine, Division of Rheumatology, Medical University Graz, Austria; 2 Clinical Institute of Medical and Laboratory Diagnostics, Medical University Graz, Austria; 3 Department of Internal Medicine, Division of Oncology, Medical University Graz, Austria; 4 Department of Internal Medicine, Diabetes and Metabolism Clinic, Medical University Graz, Austria; 5 Department of Internal Medicine, Landeskrankenanstalten Salzburg, Salzburg, Austria

## Abstract

An association between susceptibility to rheumatoid arthritis (RA) and a common -168A>G polymorphism in the *MHC2TA *gene with differential major histocompatibility complex (MHC) II molecule expression was recently reported in a Swedish population. The objective of the present study was to replicate this finding by examining the -168A>G polymorphism in an Austrian case–control study. Three hundred and sixty-two unrelated RA cases and 351 sex-matched and age-matched controls as well as 1,709 Austrian healthy individuals were genotyped. All participants were from the same ethnic background. Genotyping was performed using 5' allelic discrimination assays. The association between susceptibility to RA and the -168A>G single nucleotide polymorphism was examined by chi-square test. Comparison was made assuming a dominant effect (AG + GG genotypes versus AA genotype). In contrast to the primary report, the frequency of *MHC2TA *-168G allele carriers was not significantly different between patients and controls in the Austrian cohort. The homozygous *MHC2TA *-168 GG genotype was more frequent in matched controls than in Austrian RA patients. There was no association between the presence of RA-specific autoantibodies and the *MHC2TA *-168 GG genotype. In this cohort of Austrian patients, no association between the *MHC2TA *polymorphism and RA was found.

## Introduction

Rheumatoid arthritis (RA) is a chronic, progressive, autoimmune disease characterized by inflammation of the synovium and subsequent joint destruction [1]. It is a complex disease, with genetic and environmental factors contributing to its etiology. The prevalence of RA in Europe ranges from 0.3% to 1.6%, with higher prevalence in the north of Europe [2]. Studies in twins suggest that the genetic component of RA accounts for approximately 60% of disease susceptibility [3].

The concept of a strong genetic component to susceptibility to RA is well established, and the HLA-DRB1 locus is estimated to account for approximately 30% [4]. Several HLA-DRB1 alleles encoding the 'shared epitope' are recognized as disease-risk alleles or disease-severity alleles [5-7]. In addition, a strong association between RA-specific autoantibodies and *PTPN22 *has been reported [8,9]. Several genome-wide linkage studies and numerous association studies involving positional and/or functional candidate genes have tried to identify further RA susceptibility loci, with controversial results in different populations [10,11].

Swanberg and colleagues recently reported an association of a common -168A>G polymorphism in the *MHC2TA *gene with differential major histocompatibility complex (MHC) II molecule expression and susceptibility to diseases with inflammatory components, such as multiple sclerosis or RA [12]. Although the results of that study were convincing and biologically plausible, the need for replication of this association has been raised [13].

The aim of this study was therefore to validate and extend the reported association of the *MHC2TA *-168A>G polymorphism with RA in a group of 373 Austrian Caucasian RA cases and 373 healthy Austrian Caucasian controls.

## Materials and methods

A total of 362 RA patients and 351 healthy controls, all Caucasians, were included in the study after informed consent and approval by the local ethics committee were obtained. All patients fulfilled the American College of Rheumatology (ACR) criteria for the diagnosis of RA [14]. As an additional control group, we analyzed 1,709 healthy Austrian individuals from the population-based Salzburg Atherosclerosis Prevention Program in Subjects at High Individual Risk screening study [15,16]. All participants gave their written consent to be included in the study.

Demographic and genetic data of controls and patients, including the stage of disease as defined by the radiologic stage (Steinbroker) [17]), are summarized in Tables 1 and 2.

The *MHC2TA *genotypes were determined by a TaqMan™ fluorogenic 5'-nuclease assay (Applied Biosystems, Vienna, Austria) using Applera's Assays-by-Design custom service. The sequences of the primers and probes were as follows: forward primer, 5'-TCTTCACCAAATTCAGTCCACAGT-3'; reverse primer, 5'-ACCTCTAATTTTACCACACTCCCTTA-3'; A-probe, 5'-VIC-CCCTCCCTACACCTC-NFQ-3'; and G-probe, 5'-FAM-CTCCCCACACCTC-NFQ-3'.

Commercially available kits were used for the detection of anti-cyclic-citrullinated peptide (aCCP) (Immunoscan RA; Euro Diagnostica, Malmö, Sweden) and rheumatoid factor (AutostatTMII RF IgM; Hycor Biomedical, Kassel, Germany). The ELISA was performed according to the manufacturer's instructions.

Statistical analysis was performed using SPSS for Windows, release 11.0.1 (SPSS Inc., Chicago, IL, USA). Metric values were analyzed by Student's *t *test and are presented as the mean ± standard deviation. Categorical values were compared by chi-square test. According to methods of the primary report for the *MHC2TA *polymorphism [12], comparisons were made assuming a dominant effect (AG + GG genotypes versus AA genotype). Odds ratios (OR) and 95% confidence intervals (CI) were determined by logistic regression analysis. The criterion for statistical significance was *P *< 0.05.

## Results

*MHC2TA *genotypes were successfully determined in 362 patients and in 351 controls. Genotype distribution did not deviate from the Hardy-Weinberg equilibrium in patients or controls.

In contrast to the primary working hypothesis, the frequencies of *MHC2TA *-168 G allele carriers were not significantly different between patients and controls (Table 1). The *MHC2TA *-168 G allele was not associated with an increased risk for RA in a recessive model (GG + GA genotypes versus AA genotype; OR = 0.93, 95% CI = 0.69–1.25) or a co-dominant model (GG versus genotype GA versus genotype AA genotype; OR = 0.86; 95% CI = 0.69–1.09). The homozygous *MHC2TA *-168 GG genotype was more frequent in matched controls than in patients, and the resulting OR of the GG genotype for RA was 0.58 (95% CI = 0.34–0.99).

There was no significant difference in the genotype frequency between the RA patients and the additional larger control group (*n *= 1709) for the dominant effect (AA genotype versus AG + GG genotypes; *P *= 0.608, OR = 0.942, 95% CI = 0.751–1.151), as well as for the recessive effect (GG genotype versus AG + AA genotypes; *P *= 0.167, OR = 0.726, 95% CI = 0.462–1.141).

The *MHC2TA *gene among Austrian RA patients was not significantly associated with rheumatoid factor positivity, with an aCCP antibody titer stage of RA, or with the age at onset of the disease (Table 2).

In the present study, *MHC2TA *genotype and allele frequencies of the population differed statistically significantly from those of the primary report [12] (Table 3). The AA genotype was significantly less frequent among Austrian patients compared with Swedish patients (Table 3; *P *= 0.02). The AA genotype frequencies were furthermore lower in Austrian matched controls and in Austrian additional controls compared with the respective Swedish populations (*P *< 0.001).

Interestingly, the GG genotype frequency was significantly higher in the Austrian controls compared with the Swedish controls: 10.5% of the Austrian matched controls but only 4.8% of the Swedish matched controls carried the GG genotype (*P *< 0.001). This difference was also confirmed in both extended control groups (8.5% versus 5.1%, respectively; *P *< 0.001).

## Discussion

In the current study 362 Austrian patients with RA and their matched controls were genotyped for a single nucleotide polymorphism in the *MHC2TA *gene, which was recently reported to be associated with RA in a Swedish population [12].

The results were surprising. In contrast to the Swedish data reported by Swanberg and colleagues [12], the present study found no association between the *MHC2TA *single nucleotide polymorphism and the Austrian RA patients. To eliminate a possible bias in our matched controls we additionally genotyped a larger control group, but the genotype frequency was similar.

The reason for the difference between results of the present study and those of Swanberg and colleagues is currently unknown. In the primary report, an OR of 1.29 was observed for carriage of a *MHC2TA *-168 G allele. The present study had a statistical power of 0.40 and 0.77 to detect ORs of 1.29 and 1.5, respectively, for carriage of this allele. We are aware that this low power is a major limitation of the present study. Further larger studies are necessary to draw firm conclusions on the role of the *MHC2TA *polymorphism for RA.

Although the *MHC2TA *-168A>G polymorphism has convincingly been associated with *MHC2TA *gene expression, it is unclear whether the polymorphism itself is functional or whether it is in linkage disequilibrium with one or more causal genetic variants. If the latter was true, than this linkage disequilibrium may differ across different ethnic populations. Our results, which are in clear contrast to those previously published, could therefore be due to differences in linkage disequilibrium of the -168A>G polymorphism with the hypothesized causal variant or due to a different haplotype structure in the population of the present study. This difference may be also reflected by the fact that the prevalence of RA is not uniform across Europe, showing higher prevalences in Northern Europe compared with Southern European countries [2].

Interestingly, the frequency of the *MHC2TA *-168A allele was higher in controls from Austria compared with controls of the primary report from Sweden [12]. Different allele frequencies between Northern Europe and Southern Europe have been described for a variety of gene polymorphisms [18,19] and reflect ethnic differences across Europe. Similar to our report, a recent publication by Akkad and colleagues was unable to replicate the described association between RA and the *MHC2TA *allotype in a German population [20]. The frequency of the *MHC2TA *G allele in that study was 0.27, which is comparable with the frequency of 0.29 among combined controls of the present study. Further studies analyzing additional polymorphisms in the *MHC2TA *gene might help to elucidate the role of this gene for RA.

The lack of correlation between the presence of the specific RA autoantibodies aCCP or rheumatoid factor with *MHC2TA *alleles in our cohort is a further indication that the *MHC2TA *genotype might not be directly associated with RA. A strong association between RA-specific autoantibodies and certain single nucleotide polymorphisms has recently been reported, especially between *PTPN22 *and rheumatoid factor and aCCP [8,9]. Patients with the RA-specific allele *PTPN22 *were therefore significantly more often positive for rheumatoid factor or aCCP compared with controls [8]. Moreover, all patients who had the combination of aCCP and *PTPN22 *T polymorphism developed RA [9].

## Conclusion

We did not find any association of the *MHC2TA *-168G>A polymorphism with RA or RA-specific autoantibodies in an Austrian population. Further independent studies in other ethnic cohorts are necessary to delineate whether the differences between our results and those obtained from the Swedish population are due to ethnic bias or because of the small sample size.

## Abbreviations

aCCP = anti-cyclic-citrullinated peptide; CI = confidence intervals; ELISA = enzyme-linked immunosorbent assay; OR = odds ratios; RA = rheumatoid arthritis.

## Competing interests

The authors declare that they have no competing interests.

## Authors' contributions

BY-B and PK conceived of the study, and participated in its design and coordination and the acquisition of data. WR, WM, TCW, BP and MG carried out the molecular genetic study. WR and UL performed the statistical analysis. WG and TM participated in coordination and interpretation of the data. KB and KW participated in the acquisition of data and helped draft and revise the manuscript. H-PB was involved in drafting and revising the manuscript for important intellectual content and provided final approval of the version to be published. All authors read and approved the final manuscript.

## Figures and Tables

**Table 1:** Demographic and genetic data of Austrian rheumatoid arthritis patients and matched control subjects

	Patients (*n *= 362)	Controls (*n *= 351)	*P *value
Male gender (*n *(%))	70 (19.3)	70 (19.9)	n.s.
Age (years ± SD)	59 ± 13	59 ± 11	n.s.
Disease duration (years ± SD)	9 ± 5	-	-
Erosive disease (%)	76	-	-
DMARDs (*n *± SD)	6 ± 2	-	-
*MHC2TA *-168 AA genotype (*n *(%))	184 (50.8)	172 (49.0)	-
*MHC2TA *-168 AG genotype (*n *(%))	155 (42.8)	142 (40.5)	0.90 (AG genotype versus AA genotype)
*MHC2TA *-168 GG genotype (*n *(%))	23 (6.4)	37 (10.5)	0.044 (GG genotype versus AA + AG genotypes)
*MHC2TA *-168G frequency	0.278	0.308	0.21

DMARDS, disease-modifying antirheumatic drugs; n.s., not significant; SD, standard deviation.

**Table 2:** *MHC2TA *-168A>G genotype and clinical characteristics of Austrian rheumatoid arthritis patients

	AA genotype	AG genotype	GG genotype	*P *value	
				Dominant, AA:AG + GG genotypes	Recessive, AA + AG:GG genotypes

Rheumatoid factor (*n *(%))				0.23	0.27
Positive	136	107	14		
Negative	42	42	8		
Anti-cyclic-citrullinated peptide (*n *(%))				0.89	0.38
Positive	61	57	7		
Negative	32	29	6		
Stage (*n *(%))				0.15	0.40
1	37	40	7		
>1	138	107	15		
Age at onset of rheumatoid arthritis (years ± SD)	50 ± 16	50 ± 16	52 ± 12	0.90	0.50

SD, standard deviation. Data for rheumatoid factor were available from 349 patients, data for the clinical stage of the disease from 344 patients and data for the anti-cyclic-citrullinated peptide were available from 192 patients. The stage of disease is defined by the radiologic stage (Steinbroker).

**Table 3:** MHC2TA genotype and allele frequencies in the Austrian and Swedish study populations

	Austrian cohort			Swedish cohort [12]		
	RA patients (*n *= 362)	Matched controls (*n *= 351)	Additional controls (*n *= 1,709)	RA patients (*n *= 1262)	Matched controls (*n *= 704)	Additional controls (*n *= 1,599)

*MHC2TA *-168 AA genotype	184 (50.8)*	172 (49.0)^†^	894 (52.3)^‡^	728 (57.7)	449 (63.8)	989 (61.9)
*MHC2TA *-168 AG genotype	155 (42.8)	142 (40.5)	669 (39.1)	451 (35.7)	221 (31.4)	528 (33.0)
*MHC2TA *-168 GG genotype	23 (6.4)	37 (10.5)	146 (8.5)	83 (6.6)	34 (4.8)	82 (5.1)
*MHC2TA *-168G allele frequency	0.278	0.308	0.281	0.244	0.205	0.216

The *MHC2TA *genotype and allele frequencies of the present study population and the primary report by Swanberg and colleagues [12]. Carriers of a -168G allele were less frequent in the Austrian cohort compared with the Swedish cohort. *Austrian rheumatoid arthritis (RA) patients versus Swedish RA patients, *P *= 0.02, ^†^Austrian matched controls versus Swedish matched controls, *P *< 0.001, ^‡^Austrian additional controls versus Swedish additional controls, *P *< 0.001.
